# ALSgeneScanner: a pipeline for the analysis and interpretation of DNA sequencing data of ALS patients

**DOI:** 10.1080/21678421.2018.1562553

**Published:** 2019-03-05

**Authors:** Alfredo Iacoangeli, Ahmad Al Khleifat, William Sproviero, Aleksey Shatunov, Ashley R. Jones, Sarah Opie-Martin, Ersilia Naselli, Simon D. Topp, Isabella Fogh, Angela Hodges, Richard J. Dobson, Stephen J. Newhouse, Ammar Al-Chalabi

**Affiliations:** 1 Department of Biostatistics and Health Informatics, Institute of Psychiatry Psychology and Neuroscience, King’s College London, London, UK;; 2 Department of Basic and Clinical Neuroscience, Maurice Wohl Clinical Neuroscience Institute, King’s College London, London, UK;; 3 UK Dementia Research Institute, King’s College London, London, UK;; 4 Department of Neurology and Laboratory of Neuroscience, IRCCS Istituto Auxologico, Milan, Italy;; 5 Farr Institute of Health Informatics Research, UCL Institute of Health Informatics, University College London, London, UK;; 6 National Institute for Health Research (NIHR) Biomedical Research Centre and Dementia Unit at South London and Maudsley NHS Foundation Trust, King’s College London, London, UK;; 7 Department of Neurology, King’s College Hospital, London, UK

**Keywords:** *ALS*, *genomics*, *NGS*, *bioinformatics*, *genome analysis*

## Abstract

Amyotrophic lateral sclerosis (ALS, MND) is a neurodegenerative disease of upper and lower motor neurons resulting in death from neuromuscular respiratory failure, typically within two years of first symptoms. Genetic factors are an important cause of ALS, with variants in more than 25 genes having strong evidence, and weaker evidence available for variants in more than 120 genes. With the increasing availability of next-generation sequencing data, non-specialists, including health care professionals and patients, are obtaining their genomic information without a corresponding ability to analyze and interpret it. Furthermore, the relevance of novel or existing variants in ALS genes is not always apparent. Here we present ALSgeneScanner, a tool that is easy to install and use, able to provide an automatic, detailed, annotated report, on a list of ALS genes from whole-genome sequencing (WGS) data in a few hours and whole exome sequence data in about 1 h on a readily available mid-range computer. This will be of value to non-specialists and aid in the interpretation of the relevance of novel and existing variants identified in DNA sequencing data.

## Introduction

Amyotrophic lateral sclerosis (ALS) is a progressive neurodegenerative disease, typically leading to death within 2 or 3 years of first symptoms. Many gene variants have been identified that drive the degeneration of motor neurons in ALS, increase susceptibility to the disease or influence the rate of progression (1). The ALSoD webserver (2) lists more than 120 genes and loci which have been associated with ALS, although only a subset of these have been convincingly shown to be ALS-associated (3), demonstrating one of the challenges of dealing with genetic data interpretation of findings. Next-generation sequencing provides the ability to sequence extended genomic regions or a whole-genome relatively cheaply and rapidly, making it a powerful technique to uncover the genetic architecture of ALS (4). However, there remain significant challenges, including interpreting and prioritizing the found variants (5) and setting up the appropriate analysis pipeline to cover the necessary spectrum of genetic factors, which includes expansions, repeats, insertions/deletions (indels), structural variants and point mutations. For those outside the immediate field of ALS genetics, a group that includes researchers, hospital staff, general practitioners, and increasingly, patients who have paid to have their genome sequenced privately, the interpretation of findings is particularly challenging.

The problem is exemplified by records of *SOD1* gene variants in ALS. More than 180 ALS-associated variants have been reported in *SOD1* (2). In most cases, the basis of these variants being attributed to ALS is simply that they are rare and found in *SOD1*. Neither of these is sufficient for such a statement to be made. The p.D91A variant, for example, reaches polymorphic frequency in parts of Scandinavia, and yet has been convincingly shown to be causative of ALS. A few variants have been modeled in transgenic mice, shown to segregate with disease or have other strong evidence to support their involvement (6–10) but most do not have such support. Rare variation can be expected to occur by chance, and its existence in a gene is not evidence of relationship to a disease, making interpretation of sequencing findings difficult. Although various tools are available to predict the pathogenicity of a protein-changing variant, they do not always agree, further compounding the problem.

We, therefore, developed ALSgeneScanner, an ALS-specific framework for the automated analysis and interpretation of DNA sequencing data. The tool is targeted for use by a wide audience which includes people with knowledge outside genetics.

## Materials and methods

**Figure 1.**
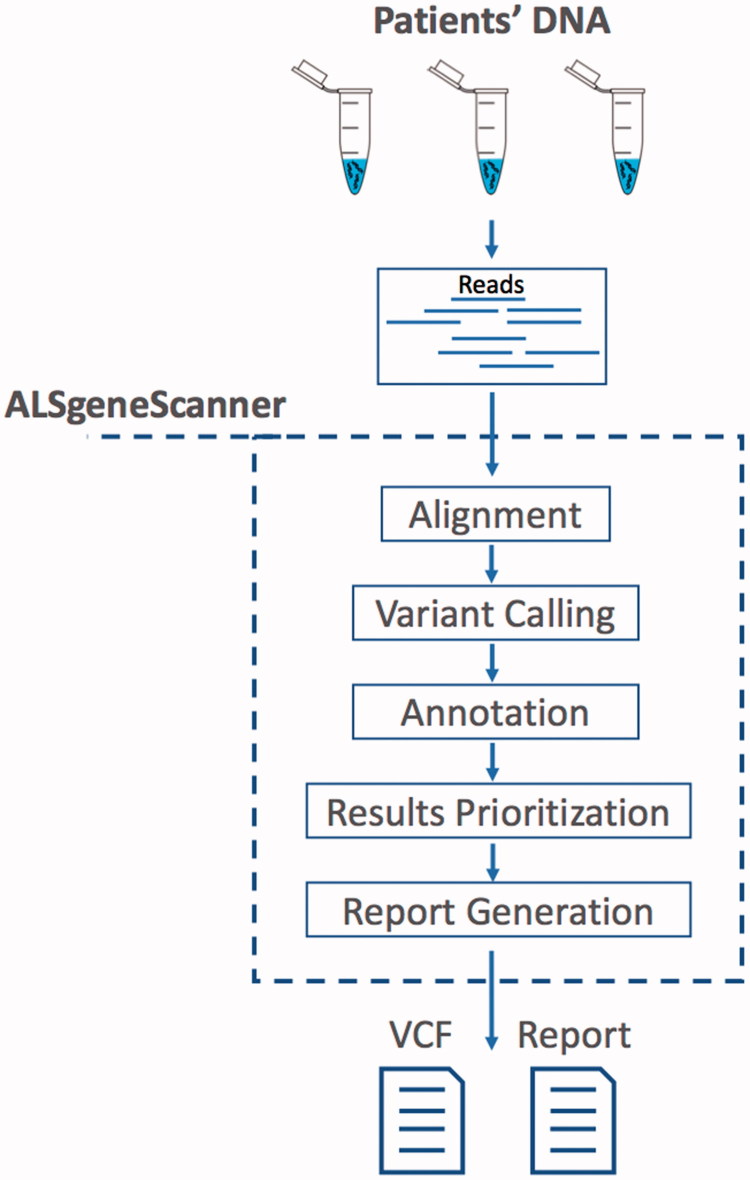
ALSgeneScanner pipeline main steps. From sequencing data in fastq format to the report generation of the results.

ALSgeneScanner is part of the DNAscan suite (11). Figure 1 shows the pipeline main steps. The pipeline accepts sequencing data in fastq and bam formats as well as DNA variants in vcf format. In the latter case, only the annotation, variant prioritization, and report generation steps are performed. A detailed description and benchmark of its analysis components have been previously published (11). ALSgeneScanner uses, among others, Hisat2 (12) and BWA-mem (13) to align the sequencing data to a reference genome, Freebayes (14) and GATK Haplotype Caller (15) to call SNVs and small indels, Manta (16) and ExpansionHunter (17) for the detection of large structural variants (bigger than 50 bps) and repeat expansions.

### Software

ALSgeneScanner is available on GitHub (18) (https://github.com/KHP-Informatics/ALSgeneScanner). The repository provides detailed instructions for tool usage and installation. A bash script for an automated installation of the required dependencies is also provided as well as Docker (19) and Singularity (20) images for a fast and reliable deployment. A Google spreadsheet with the complete list of genes and loci used by ALSgeneScanner is publicly available to visualize and comment (see GitHub repository).

### Gene and loci prioritization

ALSgeneScanner groups genes and loci associated with ALS into three classes: i) genes and loci identified by our manual scientific literature review to be associated with the disease or an influence on the phenotype in ALS (see Table 1), ii) genes in which variants of clinical significance have been reported on ClinVar (51) and for which no contradictory interpretation is present, and iii) genes for which any association evidence has been submitted to ALSoD (2). The union of these three sets of genes (available on GitHub) is used to restrict the genome analysis. However, ALSgeneScanner allows the user to use a custom list of genes.

**Table 1.** List of *ALS* genes identified by literature review.

Gene	Associated ND	Phenotype influence	Key reference
*ANG*	ALS/PD		(21)
*ANXA11*	ALS		(22)
*APOE*		Longer survival	(23)
*ATXN2*	ALS		(24)
*CAMTA1*		Shorter survival	(25)
*C21orf2*	ALS		(26)
*C9orf72*	FTD/ALS	Primarily bulbar onset	(10)
*CCNF*	FTD/ALS		(27)
*CHCHD10*	FTD/ALS		(28)
*DAO*	ALS		(29)
*DCTN1*	ALS		(30)
*EPHA4*		Longer survival	(31)
*FIG4*	ALS		(32)
*FUS*	FTD/ALS	Early age of onset and shorter survival	(9)
*HNRNPA1*	ALS		(33)
*IDE*		Shorter survival	(25)
*KIF5A*	ALS		(34)
*MATR3*	ALS		(35)
*MOBP*	ALS		(26)
*NEK1*	ALS		(36)
*NIPA1*	ALS		(37)
*OPTN*	ALS		(38)
*PFN1*	ALS	Limb-onset	(39)
*PGRN*	FTD/ALS		(40)
*SARM1*	ALS		(26)
*SCFD1*	ALS		(26)
*SOD1*	ALS	Limb-onset, early age of onset and shorter survival	(41)
*SPG11*	ALS		(38)
*SQSTM1*	FTD/ALS		(42)
*SETX*	ALS		(43)
*TAF15*	ALS		(44)
*TARDBP*	FTD/ALS		(45)
*TBK1*	ALS		(26)
*TUBA4A*	FTD/ALS		(46)
*UBQLN2*	FTD/ALS		(47)
*UNC13A*	ALS	Shorter survival	(26)
*VAPB*	ALS		(48)
*VCP*	FTD/ALS		(49)
*8p23.2*	ALS		(26)
*1p34-rs3011225*		Late age of onset	(50)

### Manual scientific literature review

The literature review was performed using several databases, including PubMed, MEDLINE, and EMBASE, to identify all articles reporting the contribution of genetic variations to the development of the disease or the modification of the phenotype in ALS from 1993, when *SOD1* was the first gene discovered to cause ALS (41), until the date of the last manuscript revision. Review articles were discarded. The resulting list of genes and loci was filtered by keeping only the ones for which the link with ALS was shown in at least two independent studies (e.g. *SOD1, FUS, C9orf72*, etc.) or cohorts (e.g. *KIF5A*), or whose variants passed the genome-wide significance threshold in GWAS studies (e.g. CAMTA1). In the latter case, if a replication study was not yet available, to avoid spurious associations, we also required that these variants were surrounded by proxies in tight linkage disequilibrium (LD) that clearly indicated the presence of an associated haplotype block. The resulting list of ALS genes and loci is kept up to date by reviewing new articles as they become available. This list, as well as the complete list of reviewed articles, is available on GitHub (https://github.com/KHP-Informatics/ALSgeneScanner).

### Variant prioritization

The pathogenicity prediction programs, SIFT (52), PolyPhen-2 HDIV and PolyPhen-2 HVAR (53), LRT (54), MutationTaster (55), MutationAssessor (56), Fathmm (57), PROVEAN (58), Fathmm-MKL coding (59), MetaSVM (60), and CADD (61) are used to prioritize variants. A variant is scored X where X is equal to the number of tools which predict it to be pathogenic. A higher priority is given to variants which are reported to be “likely pathogenic” or “pathogenic” on ClinVar. For each tool, we used the authors’ recommendations for the categorical interpretation of the variants. For each variant, the score ranges between 0 and 11 according to the number of computational tools (11 in total) that predict it to be pathogenic. In order to leave the user free to customize the prioritization criteria, both our cumulative score and the categorical variant interpretations from the 11 tools are included in the final results.

### Whole-genome sequencing

The whole-genome sequencing (WGS) sample used to assess the computational performance of ALSgeneScanner was sequenced as part of Project MinE (62). Venous blood was drawn from patients and controls and genomic DNA was isolated using standard methods. DNA integrity was assessed using gel electrophoresis. Samples were sequenced using Illumina’s FastTrack services (San Diego, CA) on the Illumina Hiseq 2000 platform. Sequencing was 100 bp paired-end performed using PCR-free library preparation, and yielded ∼40x coverage across each sample.

### Whole-exome sequencing

To assess the computational performance of ALSgeneScanner we also used the Illumina Genome Analyzer II whole exome sequencing of NA12878 (ftp://ftp-trace.ncbi.nih.gov/1000genomes/ftp/technical/working/20101201_cg_NA12878/NA12878.ga2.exome.maq.raw.bam).

### VariBench and ClinVar datasets

To assess our variant prioritization approach, we used a set of non-synonymous variants from the VariBench dataset (63) for which the effect is known and all ALS-associated non-synonymous variants stored in ClinVar (71 benign and 121 pathogenic). The VariBench variants are not ALS genes specifically, but because they are all annotated depending on whether or not they are deleterious, the general principles of the method could be tested. The dataset includes VariBench protein tolerance dataset 1 (http://structure.bmc.lu.se/VariBench/tolerance_dataset1.php) comprising 23,683 human non-synonymous coding neutral SNPs and 19,335 pathogenic missense mutations (64). None of the tools used in our pathogenicity score were trained on the VariBench dataset. However, it is possible that some VariBench variants were present in the training datasets. In order to minimize the overlap between training and evaluation sets, we derived a subset of variants (VariBenchFiltered) from the VariBench dataset by filtering out its overlap with HumVar (53), the CADD training dataset (61) and ExoVar (65), which are commonly used to train the tools (66). The resulting dataset comprising 5051 pathogenic and 14,077 neutral variants, was balanced by randomly subsampling 5051 neutral variants.

### Evaluation of performance

Receiver operating characteristic (ROC) curves and their corresponding area under the curve (AUC) statistic were calculated using easyROC (67). Accuracy, precision, and sensitivity are defined as in equation below where $T_{p}$ is true positives, $F_{p}$ false positives, $F_{n}$ false negatives, and $T_{n}$ true negatives.

$$\text{Precision}=\;\frac{T_{p}\;}{T_{p}+\;F_{p}};\text{ Sensitivity}=\frac{T_{p}\;}{T_{p}+\;F_{n}};\text{Accuracy}=\frac{T_{p}+T_{n}}{T_{p}+T_{n}+F_{n}+F_{p}}$$

### Hardware

All tests were performed on a single, mid-range, commercial computer with 16GB RAM and an Intel i7-670 processor.

### Output

Resulting variants are reported in a tab-delimited format to favor practical use of worksheet software such as iWork Number, Microsoft Excel, or Google Spreadsheets.

## Results

**Figure 2.**
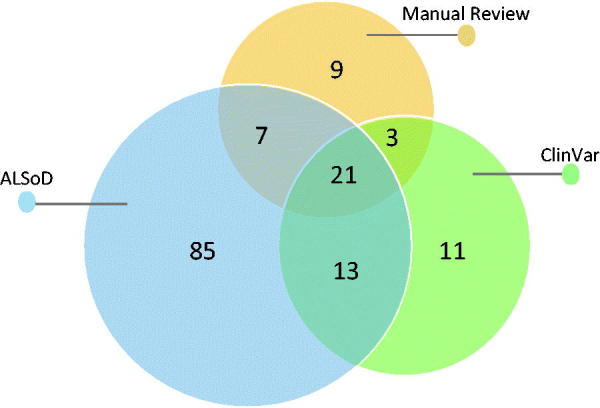
Venn diagram of the ALS related genes that we selected in our literature review, found in the ALSoD webserver and in the ClinVar database.

Manual literature review identified 486 articles describing a total of 127 genes and loci associated with ALS (the article and gene lists are available on GitHub), from which 38 genes and 2 loci (Table 1) with strong and reproducible supporting evidence of association with ALS or influence on phenotype were included. ClinVar reported SNVs and small indels in 44 genes and 4 structural variants ranging in size from 3 to 50 million base pairs. ALSoD reported variants in 126 genes and loci. The union of these sets of genes contained 149 genes and loci. The Venn diagram in Figure 2 shows the overlap between the three sets.

**Figure 3.**
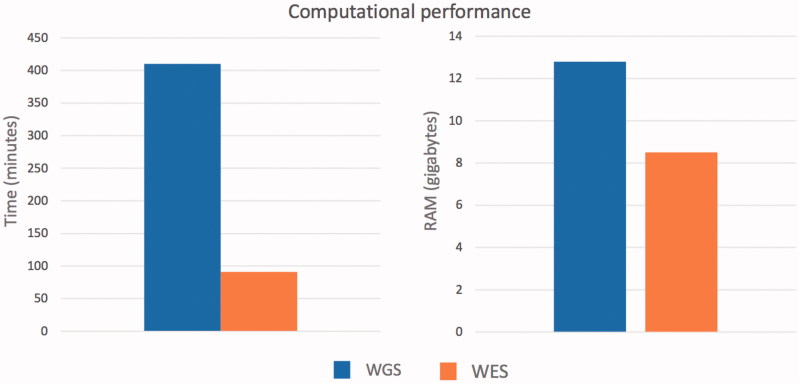
Computational performance of the pipeline to process whole-genome sequencing and whole exome sequencing data from fastq file to the generation of the final result report.

Using a midrange commercial computer (4 CPUs and 16 gigabytes of RAM) (Figure 3) ALSgeneScanner could analyze 40x WGS data of one individual in about 7 h using 12.8GB of RAM, and whole-exome sequencing data in 1 h and 20 min using 8.5GB of RAM.

**Figure 4.**
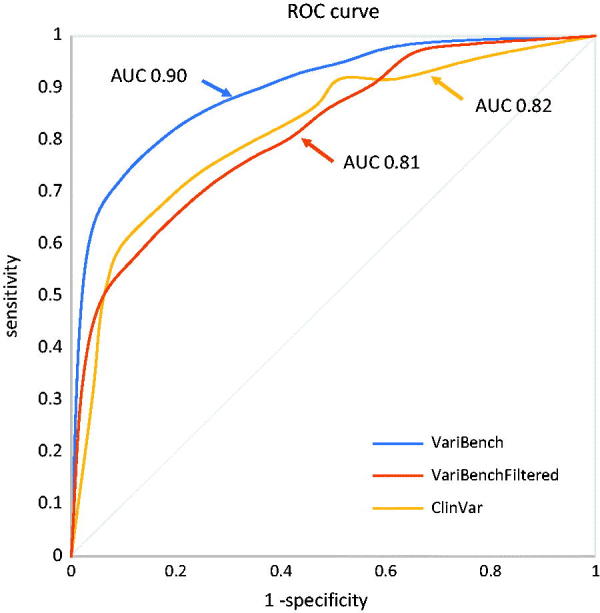
ROC curve of the performance of ALSgeneScanner on the three datasets.

We tested the computational score that the tool used to rank variants on three datasets. The VariBench dataset, the VariBenchFiltered dataset, and on the ALS associated ClinVar entries. Figure 4 shows the results on the three datasets and Table 2 precision, sensitivity and accuracy of the method in function of the chosen threshold. The ROC curve for the VariBench and VariBenchFiltered dataset (Figure 4, AUC = 0.90 and 0.81) suggests a cutoff equal to 9 which maximizes the accuracy (0.83 and 0.73) however, a lower or higher cutoff can be chosen to reach a better precision or sensitivity according to the user’s needs. For example, for diagnostics a higher sensitivity is generally required and a cutoff equal to 5 would increase the sensitivity to 0.90 (Table 2). The ROC curve for the ClinVar variants suggests a cutoff equal to 7. The AUC for such variants is 0.82 (Figure 4) and the accuracy for the ideal cutoff is 0.75 (Table 2). The better performance on the VariBench dataset can be partially explained by the fact that some of its variants were used for training the tools used by our cumulative score. However, other factors can contribute to the performance drop on the VariBenchFiltered and ClinVar ALS datasets: first the uncertainty in the ClinVar entries. ClinVar provides the community with an infrastructure to allow researchers to store their clinical observations, but the quality checks are very limited and the only filter we have adopted in this study to select the variants was the absence of contradictory entries. A similar effect is also likely for the VariBenchFiltered dataset. Indeed, filtering out all variants present in the other datasets might increase the proportion of misclassified variants. Also, the different definitions of pathogenicity and neutrality used in the different benchmark/training datasets could contribute to this effect (66). The second is the difficulty that available computational tools have in assessing the effect of ALS related variants (3,36), in part because the mechanism of ALS is unknown, and in part because at least some of the variants result in a toxic gain of function that is difficult to understand or model.

**Table 2.** ALSgeneScanner variant prioritization performance.

	VariBench			VariBenchFiltered			ClinVar ALS variants		
Score	Precision	Sensitivity	Accuracy	Precision	Sensitivity	Accuracy	Precision	Sensitivity	Accuracy
0	0.430	1	0.430	0.500	1	0.500	0.612	1	0.613
1	0.507	0.990	0.581	0.560	0.984	0.606	0.659	0.957	0.670
2	0.549	0.978	0.644	0.592	0.968	0.651	0.707	0.949	0.728
3	0.580	0.950	0.682	0.609	0.907	0.663	0.745	0.949	0.770
4	0.618	0.928	0.721	0.634	0.860	0.682	0.754	0.889	0.754
5	0.653	0.900	0.751	0.657	0.804	0.693	0.798	0.812	0.759
6	0.692	0.875	0.779	0.687	0.766	0.708	0.832	0.761	0.759
7	0.736	0.841	0.801	0.721	0.719	0.720	0.863	0.701	0.749
8	0.783	0.796	0.817	0.762	0.657	0.726	0.911	0.615	0.728
9	0.845	0.731	0.827	0.817	0.582	0.726	0.926	0.538	0.691
10	0.919	0.635	0.819	0.895	0.486	0.714	0.931	0.462	0.649
11	0.954	0.436	0.748	0.937	0.315	0.647	0.925	0.316	0.565

Correlation analysis was performed to investigate the correlation between the 11 tools used by our score, using the categorical results of each individual tool on the VariBenchFiltered dataset. Supplementary Table 1 shows the results of this analysis. The average correlation was 45% and the standard deviation 14%. Only PolyPhen-2 HDIV and PolyPhen-2 HVAR showed a strong correlation (83%). PolyPhen-2 HDIV differs from PolyPhen-2 HVAR in the training dataset which only included Mendelian disease variants. These tools can provide the user with complementary useful information.

## Discussion

We have developed ALSgeneScanner, a fast, efficient, and complete pipeline for the analysis and interpretation of DNA sequencing data in ALS, targeted for use by a wide audience including non-geneticists. The method is able to distinguish pathogenic from nonpathogenic variants with high accuracy and reports findings in a simple format, able to be exported for further analysis. With the decreasing costs and increasing availability of next-generation sequencing, health care professionals and motivated patients are progressively more likely to have WGS data available, without the tools to interpret findings. An automated system to provide a meaningful report, therefore, has a potentially important part to play in giving patients ownership of their data and arming them with the knowledge to understand it, but this should always be interpreted with the assistance of a specialized genetic counselor.

**Figure 5.**
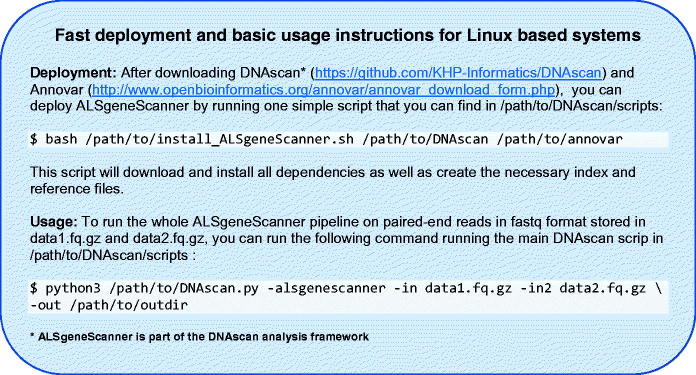
Deployment and usage instructions.

Omictools (68), a web database where available bioinformatics tools are listed and reviewed, lists over 7000 such tools for next-generation sequencing, including more than 100 pipelines; given the great interest in this field, new tools are frequently released. As a result, designing a bioinformatics pipeline for the analysis of next-generation sequencing data, keeping the system simple to use on a standard computer and translating the output into a format that is easily understood, is not trivial, and requires specialized expertise. The computational effort and the informatics skills required to use typical pipelines can dramatically limit the use of next-generation sequencing data. Adequate high-performance computing facilities and staff specialized in informatics are not always present in medical and research centers. Furthermore, the use of cloud computing facilities, which could theoretically provide unlimited resources, is not always possible due to privacy and ownership issues, cost and the expertise required for their use. To this end, ALSgeneScanner is computationally light as it can run on a midrange commercial computer. Performing the same analyses with other widely used pipelines, e.g. SpeedSeq (69) and GATK Best Practice Workflow (15), would require high-performance facilities (HPC) and about 3–10 times more computational resources than for ALSgeneScanner (11). It is easy to use since it performs sophisticated analyses using only a few command lines (see Figure 5) and is comprehensive, including the necessary analyses to identify all known ALS associated genetic factors. Finally, a tab-delimited output, in which the analysis results are enriched with information from several widely used databases such as ClinVar, ALSoD, our manual literature review, pathogenicity scores and the graphical visualization utilities (see Supplementary Material) integrated in the pipeline as part of DNAscan (11), favor an easily accessible interpretation of the results. No other currently available pipeline provides the user with such a comprehensive end-to-end analysis framework.

Our table of sensitivity, specificity, and accuracy (Table 2) means that the appropriate cutoff can be used to interrogate data, depending on whether the aim is the exclusion of potentially harmful variants, or the detection of definitely harmful variants.

ALSgeneScanner puts a powerful bioinformatics tool, able to exploit the potentialities of next-generation sequencing data in the hands of patients, ALS researchers, and clinicians.

## Supplementary Material

ALSgeneScanner_supplementary_material.docx
