# Supplementary material for: ALSgeneScanner: a pipeline for the analysis and interpretation of DNA sequencing data of ALS patients
Source: Amyotroph Lateral Scler Frontotemporal Degener. 2019 Mar 5;20(3-4):207–15. doi: 10.1080/21678421.2018.1562553 (PMC6567555; doi:10.1080/21678421.2018.1562553)
Supplement: ALSgeneScanner_supplementary_material.docx [file IAFD_A_1562553_SM9738.docx]

**Supplementary Material of ALSgeneScanner: a pipeline for the analysis and interpretation of DNA sequencing data of ALS patients**

Alfredo Iacoangeli^a,b^**^*^**, Ahmad Al Khleifat^b^, William Sproviero^b^, Aleksey Shatunov^b^, Ashley R Jones^b^, Sarah Opie-Martin^b^, Ersilia Naselli^b^, Simon D Topp^c^, Isabella Fogh^b,d^, Angela Hodges^b^, Richard J Dobson^a,e,f^, Stephen J Newhouse^a,e,f^, Ammar Al-Chalabi^c^

^a^ Department of Biostatistics and Health Informatics, King’s College London, London, UK; ^b^ Maurice Wohl Clinical Neuroscience Institute, King’s College London, London, UK; ^c^ UK Dementia Research Institute, King’s College London, London, UK; ^d^ Department of Neurology and Laboratory of Neuroscience, IRCCS Istituto Auxologico Italiano, Milan, Italy; ^e^ Farr Institute of Health Informatics Research, UCL Institute of Health Informatics, University College London, London, UK; ^f^ National Institute for Health Research (NIHR) Biomedical Research Centre and Dementia Unit at South London and Maudsley NHS Foundation Trust and King’s College London London, UK.

*Correspondence should be addressed to alfredo.iacoangeli@kcl.ac.uk

**Correlation Analysis**

Correlation analysis was performed to investigate the correlation between the 11 tools used by our score, using the categorical results of each individual tool on the VariBenchFiltered dataset. Supplementary Table 1 shows the results of this analysis. The correlation coefficients were calculated using the Microsoft Excel (version 16.15) function CORREL(). The average correlation was 45% and the standard deviation 14%. Only Polyphen2 HDIV and Polyphen2 HVAR showed a strong correlation (83%). Polyphen2 HDIV differs from Polyphen2 HVAR in the training dataset which only included Mendelian disease variants. These tools can provide the user with complementary useful information.

|  | SIFT | Polyphen2_HDIV | Polyphen2_HVAR | LRT | MutationTaster | MutationAssessor | FATHMM | PROVEAN | Fathmm MKL_coding | MetaSVM | CADD |
| --- | --- | --- | --- | --- | --- | --- | --- | --- | --- | --- | --- |
| SIFT | 1 | 0.538 | 0.565 | 0.380 | 0.403 | 0.535 | 0.241 | 0.590 | 0.395 | 0.376 | 0.538 |
| Polyphen2_HDIV |  | 1 | 0.830 | 0.442 | 0.459 | 0.503 | 0.239 | 0.504 | 0.464 | 0.393 | 0.610 |
| Polyphen2_HVAR |  |  | 1 | 0.478 | 0.496 | 0.546 | 0.268 | 0.557 | 0.481 | 0.445 | 0.607 |
| LRT |  |  |  | 1 | 0.609 | 0.394 | 0.198 | 0.429 | 0.576 | 0.315 | 0.515 |
| MutationTaster |  |  |  |  | 1 | 0.423 | 0.250 | 0.443 | 0.725 | 0.379 | 0.609 |
| MutationAssessor |  |  |  |  |  | 1 | 0.282 | 0.578 | 0.395 | 0.446 | 0.469 |
| FATHMM |  |  |  |  |  |  | 1 | 0.227 | 0.215 | 0.748 | 0.208 |
| PROVEAN |  |  |  |  |  |  |  | 1 | 0.404 | 0.375 | 0.498 |
| fathmm-MKL_coding |  |  |  |  |  |  |  |  | 1 | 0.317 | 0.629 |
| MetaSVM |  |  |  |  |  |  |  |  |  | 1 | 0.324 |
| CADD |  |  |  |  |  |  |  |  |  |  | 1 |

Supplementary Table 1: Correlation coefficients of the 11 tools used by ALSgeneScanner to predict the pathogenicity of variants.

**Literature review**

Manual literature review identified 486 articles describing a total of 127 genes and loci associated with ALS.

A list of the 486 article titles can be found at the following link:

https://docs.google.com/spreadsheets/d/1dNoquP8aSAr85X9xqPGjKnvaxpQadJ4iyXuk5hsYx3Y/edit?usp=sharing

A list of the 127 genes and loci associated with ALS can be found at the following link:

https://docs.google.com/spreadsheets/d/1uwuIrWwrl4UzVYMOJPyDQ7wTcn3LvfjJWPPRGvKfi_U/edit?usp=sharing

**Visualisation utilities**

ALSgeneScanner provides utilities for visualisation and interpretation of the results. Three iobio services are locally provided with the pipeline allowing for the visualisation of the alignment file (bam.iobio), the called variants (vcf.iobio) and for a gene based visualisation and interpretation of the results (gene.iobio, Supplementary Figure 1)

These utilities are also available for evaluation at the following links:

http://bam.iobio.io

http://vcf.iobio.io

http://gene.iobio.io


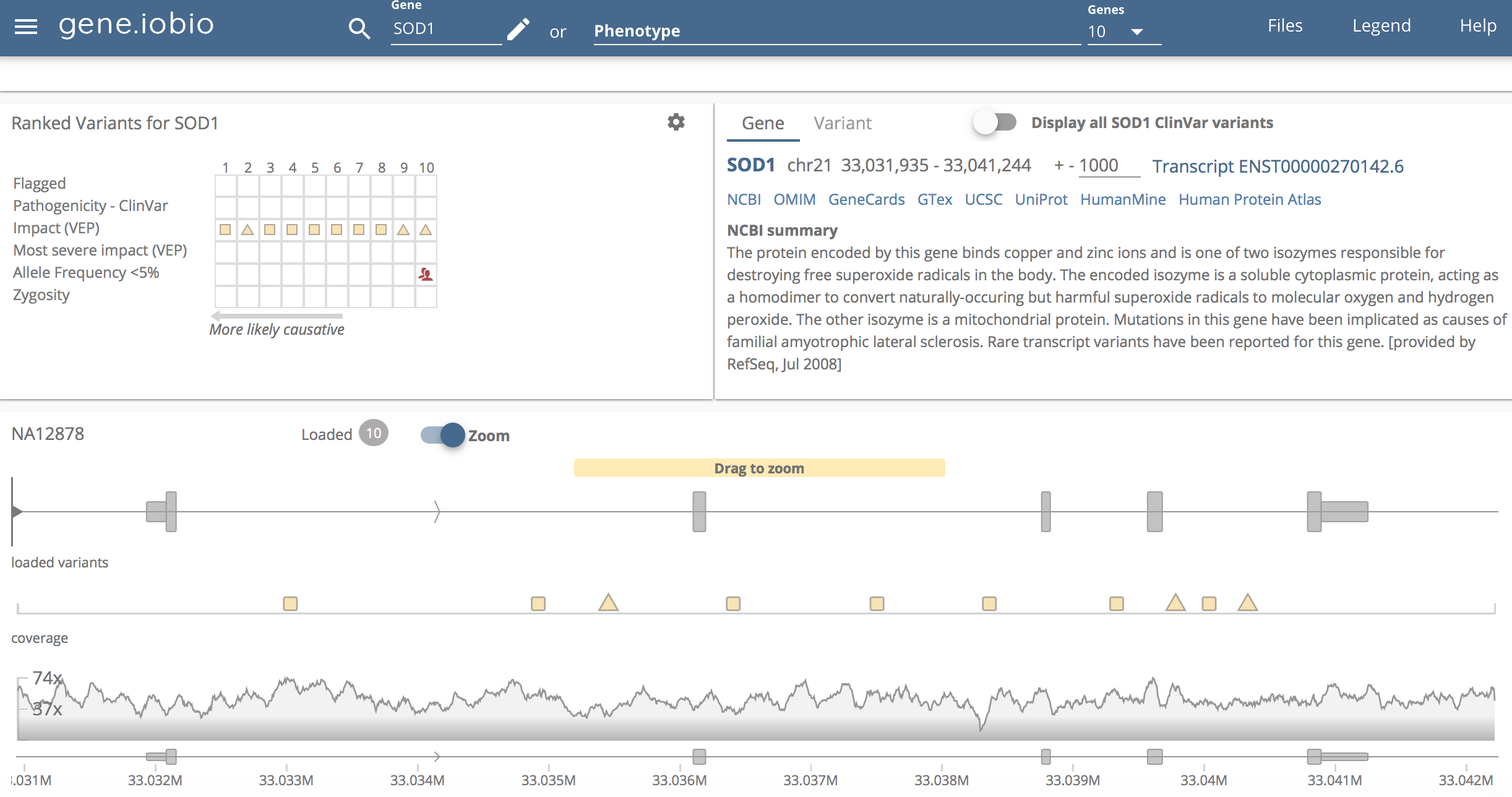


Supplementary Figure 1: Screenshot of the gene.iobio platform showing the Illumina platinum genome SOD1 variants of NA12878.
